# Premarital HIV testing in Malaysia: a qualitative exploratory study on the views of major stakeholders involved in HIV prevention

**DOI:** 10.1186/s12914-017-0120-8

**Published:** 2017-05-10

**Authors:** Sima Barmania, Syed Mohamed Aljunid

**Affiliations:** 1 International Centre for Casemix and Clinical Coding, Faculty of Medicine, National University of Malaysia, PPUKM Complex, Jalan Yaacob Latiff, Cheras, Kuala Lumpur, 56000 Malaysia; 2 United Nations University International Institute for Global Health, Kuala Lumpur, Malaysia; 3 Department of Health Policy and Management, Faculty of Public Health, Kuwait University, Hawally, Kuwait; 4 University College London, Institute of Education, London, United Kingdom

**Keywords:** HIV testing, HIV prevention, premarital, Malaysia, Islam, Human rights

## Abstract

**Background:**

HIV screening has existed in numerous methods as an important part of HIV prevention efforts over the years. Premarital HIV testing for couples who wish to marry has been implemented in a number of regions, which often operate in a mandatory rather than voluntary basis and is considered a contentious issue, with viewpoints held in favour and against. One such region is Malaysia which has a policy of mandatory premarital HIV testing of prospective Muslim married couples. The purpose of this study is to understand stakeholders’ views on premarital HIV testing given the Malaysian Islamic context.

**Methods:**

35 in-depth face to face semi-structured interviews were undertaken with key stakeholder groups involved in HIV prevention policy in Malaysia, namely, officials from the Ministry of Health, religious leaders and people living with HIV. Participants were recruited from the Klang Valley area, from July to December 2013, using purposive sampling techniques. Inclusion criteria necessitated that participants were over the age of 18 and provided full consent. Interviews were audiotaped, followed a standardised topic guide, transcribed verbatim and analysed using a framework analysis.

**Results:**

Participants identified pre-marital HIV testing as an effective HIV prevention policy implemented in Malaysia and was viewed, for the most part, as a positive initiative across all stakeholders. Religious leaders were supportive of testing as it provides a protective mechanism, in line with the teachings of the Shariah, while Ministry of Health officials considered it a normal part of their HIV prevention screening initiatives. However, there were concerns surrounding issues such as confidentiality, counselling and discrimination surrounding the test described by the PLHIV group.

**Conclusion:**

The findings of this study show that among the participants interviewed was strong support for mandatory premarital HIV testing, which could possibly expose the vulnerability to HIV, reluctance to test and other areas in the HIV response in Malaysia that need to be addressed. Furthermore, although international health organisations are vehemently against mandatory premarital HIV testing, the strong local support for such measures and the mismatch between these views is worth exploring in more detail, given the cultural, social and religious context.

## Background

Screening for HIV testing is an important part of HIV prevention efforts, along with increased education and awareness, with screening existing in numerous guises, for the safeguarding of blood products used for transfusion or during antenatal check-ups of pregnant women. Furthermore, a policy of premarital HIV testing for prospective couples who wish to marry has been implemented in a number of regions, such as parts of Africa, the Middle East and Asia, amongst Christian and Muslim populations, which are often implemented on a mandatory basis rather than in a voluntary capacity [1–6]. Thus, premarital HIV testing is considered a contentious issue with stakeholders viewing the initiative as advantageous as well as disadvantageous inciting arguments that span public health, ethical, scientific and rights, which are discussed through the course of this paper. Furthermore, although international health organisations are vehemently against mandatory premarital HIV testing, the strong local support for such measures and the mismatch between these views is worth exploring in more detail, given the cultural, social and Islamic context.

Arguments in favour of premarital HIV testing centre around the benefit to public health of such screening having the ability to diagnose HIV infection in someone not recognised as being positive [7]. It has also become an ethical debate, raising issues regarding confidentiality. For instance, Ebrahim discusses the scenario of a South African, Muslim gentleman who became HIV positive and had not informed his wife of his diagnosis prior to his marriage; later the wife discovered she was HIV positive while 8 months pregnant, the husband subsequently died of AIDS, leaving both mother and child living with HIV, dependent on family [8].

However, many of the objections to premarital HIV testing centre on human rights issues, specifically in cases where such screening is mandatory. Although international organisations such as the WHO, UNAIDS and UNHCR accept that some types of mandatory testing, such as of blood, blood products and organs, is deemed ethical and necessary, consider that all other forms of mandatory testing “is never sanctioned and opposed”. All HIV testing services should follow the ‘5 C’s’ of informed consent; confidentiality, counselling, correct test results and connection to HIV services, both of prevention and treatment [9]. Adding that that a number of conditions must be in place to ensure consent to HIV testing is truly informed which include that healthcare workers must ‘provide sufficient information in order for clients to fully understand the implications of HIV testing’. Often in mandatory premarital HIV testing, such standards are relatively hard to ensure.

The Open Society institute [1] has discussed the rising number of regions that have adopted mandatory premarital HIV testing, which include both Christian countries, such a Nigeria and Uganda and Muslim countries, such as Bahrain and Saudi Arabia. They conclude that premarital HIV testing not only compromises the principles of HIV testing but also is an infringement upon human rights, especially of the ‘right to marry’ and find a family. They regard mandatory premarital HIV testing as a fundamental ‘human rights concern’, citing Article 16 of the Universal Declaration of Human Rights and the “right of men and women of full age, without any limitation due to race, nationality or religion…to marry and to found a family”. Also inadvertently stigmatising people who may be at risk of infection, who may opt out of marriage altogether because of the fear of a positive result and the subsequent questions that could be asked by family members.

Other issues highlighted include the lack of confidentiality and cite Malaysia as an example [1] whereby Muslim couples have to ‘submit a certificate disclosing their HIV status to the state religious department when applying for a license to marry’. The right to privacy and confidentiality undermined by the number of people involved in the testing and counselling procedure, with often no clear guidelines or protocols as to who is privy to the results and where this information could be disseminated and possibly catastrophic consequences in terms of social stigma and discrimination of a breach of confidentiality.

In addition, some have argued against such policies in Asia and the Middle East, for example in India. Malhotra and colleagues argue against premarital testing for a multitude of reasons, including the increased risk of stigma and discrimination of those living with HIV, the issue of a test being within the ‘window period’, role of the state, limiting the rights of women and conclude that ultimate responsibility lies with the individual [10]. They argue that ultimately premarital HIV testing can denigrate women’s rights and disempower them as opposed to supposedly protecting them, as if both parties test negative before marrying, the woman would be less able to negotiate condom use of safe sexual practices, making them more vulnerable and at risk of HIV infection [10].

They also argue that the state’s role is to create an enabling environment to obtain information about HIV ‘conducive to voluntary counselling and testing, rather than through coercive mandatory testing strategies’ [10].

Furthermore, practical considerations such as cost effectiveness have also been raised. Tan and colleagues discuss the cost-effectiveness of HIV screening in the general population in the form of premarital HIV testing and acknowledged that there had not been any cost-effective analysis undertaken on premarital HIV testing amongst Muslim couples [11]. Adding, that the test is only useful for one point in time and does not guarantee that those tested will not be exposed in the future and can give rise to a false sense of security. Also, there are issues as to whether confidentiality can be actually kept when religious officers are involved, as a marriage is often seen, as not merely just the union of two individuals, but of two families [11].

## Premarital HIV testing in Malaysia

One country that has a policy of mandatory premarital HIV testing of prospective Muslim married couples is Malaysia [12]. Malaysia, a predominantly Muslim majority country is defined as having a concentrated epidemic amongst high-risk groups such as, Men who have Sex with Men (MSM), Sex Workers (SW) and Transgender women (TG) [13, 14]. Increasingly, sexual transmission of HIV predominates, rather than through intravenous drug users, which was the case previously, alongside a feminization of the epidemic with housewives being newly infected [14]. Malaysia is fortunate to have first line Anti Retrovirals (ARV) provided for those People Living with HIV (PLHIV) by the government, as well as provision of HIV testing although the uptake for the latter tends to be low.

Johor was the first state in Malaysia to initiate screening in 2001, with other states implementing testing at later stages. Khebir and colleagues undertook a study regarding the premarital HIV screening programme in Johor, which found 123 new cases of HIV detected (0.17%) out of 74,210 respondents, compared to antenatal screening (0.05%) [15].

The rationale for the testing is to limit the spread of HIV from spouse to spouse, or their offspring and the authors argue that it acts as an effective measure to prevent early detection of HIV and Prevention of Mother to Child Transmission of HIV (PMCT) [15]. Islamic scholar Hashim Kamali, in the early days of the implementation of the pre-marital HIV screening programme, discussed the Islamic issues relating to mandatory HIV testing and the passing of the Johor Islamic religious council fatwa that made HIV testing compulsory for all Muslim couples planning to wed in the state of Johor [16]. He specified the justification falling under the rule of *Maslaha* (public interest) and intended to protect ‘religion, life, property, intellect and lineage’ (*Hukm*) [16].

There are also other forms of HIV screening in Malaysia, namely amongst antenatal mothers which was started in 1997 as a pilot before being implemented in all government hospitals in 1998 [17]. The screening was a constituent of the Prevention of Mother to Child transmission of HIV, including ARV during pregnancy, safer delivery and feeding, however, the screening was under an ‘opt out’ approach (i.e. voluntary), with the overwhelming majority of 98% participating [18]. This is interesting because there are some researchers who argue there should be mandatory HIV testing among pregnant women whilst voluntary for premarital testing, as antenatal screening offers a greater protective advantage whilst premarital can be potentially damaging if the diagnosis, if positive, is disseminated in public [19].

Given the myriad of viewpoints of premarital HIV testing internationally, the purpose of this study is to understand stakeholder’s views in the Malaysian, Islamic context.

## Methods

This study is part of a larger research initiative, looking at the influence of Islam amongst stakeholders’ involved in HIV prevention policy in Malaysia, using both qualitative and quantitative methods. The larger project was predominantly qualitative in nature, consisting of in-depth interviews, which were face to face. However after all the qualitative interviews were completed, a smaller quantitative component was undertaken over a 4 month period from December 2013 to April 2014. This consisted of 252 self- administered questionnaires to key stakeholders groups to ascertain their knowledge of HIV.

This paper explores the qualitative component and specifically relates to the issue of pre-marital HIV testing in Malaysia as it emerged as a sub-theme of ‘HIV prevention in Malaysia’. A summary of the themes from the qualitative research are as follows: View of life and health in Islam, sex outside marriage, understandings of HIV, HIV prevention in Malaysia, use of condoms, transgender women, men who have sex with men, law and authority, stakeholder relationships, and action to be taken. The findings conclude that Islam played an important role in shaping health policies and strategies relating to HIV prevention in Malaysia.

Firstly, key stakeholder groups involved in HIV prevention policy in Malaysia were identified, including Ministry of Health officials, religious leaders and people living with HIV. Participants were recruited from the Klang Valley area (Kuala Lumpur and Putrajaya), from June until December 2013, after approval from UKM research and ethics committee. In-depth interviews were conducted with the three major groups of stakeholders identified as being involved in HIV prevention policy in Malaysia. Participants were recruited through purposive sampling techniques until saturation point was reached. Thirty-five face-to -face semi-structured interviews were undertaken, in total 11 of which were with religious leaders, 5 were Ministry of Health officials and 19 representing those living with HIV. Semi-structured interviews were adopted since they are more appropriate for the research objectives and context than unstructured interviews as they allow similar data to be collected by all respondents ensuring greater standardisation, while allowing rephrasing of the question according to participant understanding and sensitivity.

People living with HIV were identified using a long established, Malaysian non-governmental organisation who were fully briefed on the inclusion and exclusion criteria. The selected participants consisted of men who have sex with men, transgender women, sex workers and heterosexual women, all of whom were Muslim. Participants from the religious leader group included individuals from Islamic academic institutions, the Shariah Department, Department of Religious Affairs at both the national and state level (within the Klang valley region). The inclusion criteria of those that were eligible to participate consisted of both general and specific criteria, particular to stakeholder group. General criteria stipulating that participants were over the age of 18 and resided within the Klang Valley region of Peninsular Malaysia. Criteria for PLHIV stipulated that participants had to be living with HIV for 1 year or more. Participants were excluded if they were under the age of 18, or did not provide fully informed written consent. The same interviewer (SB) conducted all 35 interviews, which lasted between 60 and 90 min in duration and followed a topic guide, which ensured standardisation. The topic guide included participants’ views on HIV, perceptions of sex outside marriage, the importance of Islam, as well as HIV prevention and policies in Malaysia. Interviews were conducted in person, were digitally recorded and subsequently transcribed verbatim professionally. Interviews were conducted for the most part in English, however on occasion translation during the interview was sought from fellow colleagues. In addition, some manual notes were taken during the interview which highlighted any non-verbal communication. Religiously appropriate attire was worn by the researcher when interviewing Islamic religious leaders, including the covering of the head and limbs. A good rapport was established during the interview, ensuring the interviewee felt comfortable communicating with the interviewer whilst also being cognizant of any potential sensitivity and unease and probing accordingly.

The process of data analysis resumed early on after data collection and was conducted by the primary author (SB) who has previous experience with qualitative data collection techniques, undertaken in a thorough and comprehensive manner to ensure rigour with the stages described below.

After each interview, the audio recording was listened to again by the same interviewer (SB) to ensure vocal clarity, familiarise with, downloaded and then sent for professional transcription, which yielded a verbatim copy. This transcript was checked to the corresponding audio recording to ensure the transcript was free of mistakes, printed, read in detail, on a line by line basis. Subsequently, the transcript was read again, this time making annotations and noting potential themes as well as providing another opportunity for reflection. A collection of around 15 transcripts which spanned across all the stakeholder groups interviewed was used as a sample to design a framework analysis [20], deemed more appropriate for health policy research. This resulted in the emergence of certain themes and sub-themes, which was then used to analyse all 35 transcripts.

10 central themes emerged, with one theme relating to current HIV prevention policies in Malaysia, with the issue of premarital HIV testing as a specific sub-theme and presented according to stakeholder group.

## Results

The majority of participants interviewed for the study, identified pre-marital HIV testing as an effective current HIV prevention policy implemented by Malaysia and was seen as a positive initiative. The beliefs, opinions and concerns of participants are presented below according to the three stakeholder groups studied; namely religious leaders, PLHIV and officials from the Ministry of Health.

### Religious leaders

Amongst religious leaders who participated in the study, there was strong support for premarital HIV testing, considering that such a measure provides a solid protective mechanism.

*”This is good [pre-marital HIV testing]…to protect”. (IV 33 Religious Leader)*

The rationale behind the statement being that foremost premarital HIV testing was a protection for themselves, the prospective couple, both husband and wife “for their own safety”.

This participant believed that premarital HIV testing both protected from acquiring HIV and protecting people from passing on HIV being transmitted, from ‘*a man to a woman’*, as *‘many people do not have an education'.* Incidentally, later in the interview, the participant revealed that his own brother-in-law had died of AIDS a month previously and this event could have framed his views of pre-marital HIV testing.

Premarital HIV testing was viewed by religious leaders as a proactive way in which prospective couples could know their HIV status and thus make fully informed life decisions, including whether they wished to marry or not following the test results.

*“To me, it’s good that the people who are getting married know whether or not they have HIV. Some of them may have contracted it without knowing and it’s good that they have that information, getting married is a big thing. So you know, if they know that one of them has HIV, at least they will make the decision to get married based on that information, knowing that one of them has HIV, whether they want to go through with it [the proposed marriage], or if they want to go through with it, how should they live their lives together’. (IV 1 Religious Leader)*

This religious leader was quite atypical in the sense that he, unlike most other religious leaders, was aware of some of the opposing views of pre-marital HIV testing, issues such as human rights and possible discrimination towards people living with HIV. Adding further:

*“It’s not something that should be used as a form of discrimination…some people don’t like the idea because they think it’s like a discrimination to those with HIV…but to me, if you look at this positively, I think it’s also the right of the future spouse to know whether or not his or her husband or the wife or the future wife has HIV, because it involves basically their state of health”*

It is worthwhile highlighting that the participant himself, without the interviewer mentioning human rights, articulates and justifies his argument for premarital HIV testing within a human rights framework, within a health context.

Furthermore, many religious leaders supported premarital HIV testing, specifically as they deemed the measure as being in keeping with the teachings of the Shariah (Islamic law) as articulated in the extract below:

*“Well I think the Shariah would…would like to protect I guess, people against any kind of harm. So in that regard, I think this, it’s a right step to be taken. No, it’s not criminalising anybody but the Shariah would like to protect the individual’. (IV 35 Religious Leader)*

It is noteworthy that the participant categorically (and on his own accord), denies that mandatory testing criminalises those who are tested, given opponents, have argued that such measures could be used for nefarious purposes, criminalising or at least stigmatising those living with HIV. There was some indication of this possible stigma, articulated in this excerpt from a religious leader indicating his reasons for backing premarital HIV testing.

*”Because when someone is having a marriage, they will have a child. So we don’t want to have an HIV child”. (IV 12 Religious Leader)*

The logic behind the above statement was that premarital HIV testing essentially protected the unborn child from being born with HIV. Some religious leaders were a bit more cautious and whilst supported premarital HIV testing under the premise of “*hukum*” (protection of ‘life, heredity, lineage etc.), thought there should be a degree of privacy and confidentiality. However, where exactly the boundaries of privacy and confidentiality should be drawn remained unclear between religious leaders; some believed that as a marital union in Islam is not merely of two people, but the joining of two families, both families and the imam should be aware of the HIV status, as described below:

*“This is a very good idea, the pre-marital…but the programme… the couple are going to have a test…we doubt…the result is not sharing for the Imams and for the family, it’s not good. We do not agree.” (IV 2 Religious Leader)*

Other religious leaders believed that it was the discretion of the individual to disclose their status to whom they wish.

It was clear that religious leaders were keen to safeguard people’s future and premarital HIV testing served as a way of ensuring this, described succinctly by the following participant:

*‘Islam is actually a religion that looks to the future. This premarital HIV test, it’s actually a very good thing. Why? Because you want to prevent whatever the possible thing that might happen in the future. Because for example, if one of the bride or the bridegroom is positive of HIV, then it would be infected to the wife, to the husband, to the kids and so on.’ (IV 13 Religious Leader)*

### Ministry of health

Officials from the Ministry of Health described strong support for premarital HIV testing in Malaysia under the remit of public health, with many reiterating the sentiment described below:

*“I believe it’s [pre-marital HIV testing] a good idea.” (IV 25 Ministry of Health)*

Furthermore, the pre-marital HIV test was seen as both cost-effective and a worthy HIV prevention strategy, articulated by one participant.

*“Prevention of social disharmony, to prevent marriage disharmony, I believe it’s very cost-effective.” (IV 25 Ministry of Health)*

Adding*:*

*“In Kuala Lumpur people have been found to be HIV positive, they have decided not to go ahead with the marriage. Of course, there are instances where they know their husbands or brides to be are HIV positive, they still carry on with the intention of getting married”*

The origins of premarital HIV testing is also well described by participants and was understood to have been established gradually state by state before it became practised across the country, as described by the following participant from the Ministry of Health.

*“It was started in 2001, in the state of Johor. And after that it started scaling up in others, I mean, it spread in other states and now almost every state has the programme on pre-marital screening”. (IV 3 Ministry of Health)*

It was clear that the tests were restricted to Muslim Malay couples who wished to marry, amid rising rates in this population.

*“Initially, it was among the Muslims because our figures show that Muslims 70% of our tests are among the Muslims. But later on, in 2009 everybody who wants to get married can go for the testing”. (IV 3 Ministry of Health)*

Some participants were unsure as to which stakeholder requested the implementation of pre-marital HIV testing in Malaysia, with the consensus believing that it was prompted by the religious leaders.

*“Yeah, the religious people want that test to be done but even if I ask any people, did you know this or the rules by the religious department, they still want to get this done because every day you do that, it becomes a behaviour, it becomes a norm. Same also, now premarital testing is normal almost, norm already”. (IV 3 Ministry of Health)*

Although the initial seeds may have arisen from the religious leaders’ camp, discussions were conducted with the Ministry of Health who were in support of the initiative.

*“Pre-marital screening test, I was directly involved in it, in this project when we were discussing with the religious department. And we have to put it right that the idea of the pre-marital screening came from the religious department of Johor, from the mufti himself, looking at the issue of HIV among Muslim in Johor during, I think, 2000-2001. So he felt that the Muslim leadership come down and reach those people who are at risk of getting HIV. So it was the religious initiative. And we supported that”. (IV 31 Ministry of Health)*

The premarital HIV test is now offered to non-Muslims and is now normalised in Malay society. The following excerpt highlights the confusion on conflicting sentiments expressed by the same individual within the Ministry of Health, first insisting that the test is not compulsory or mandatory, but voluntary.

*“That’s done actually mainly for Muslim couples. For the non-Muslims, it’s not compulsory because there are different perspectives of it. But again, HIV screening has always been voluntary”. (IV 11 Ministry of Health)*

Then the same participant continued to explain that it is a requirement for other matters pertaining to religion and marriage.

*“So they have made it mandatory for other things, so there is this issue of whether it should be made a legal requirement and all but as it is the decision of the Muslim council, then all those Muslims couples who have to marry have to undergo that”. (IV 11 Ministry of Health)*

Language is used to articulate the measure in a more accepting way; so although HIV testing is not obligatory under the Ministry of Health, it is still required if you wish to marry under Ministry of Religious Affairs regulations with the HIV testing service provided under the remit of the Ministry of Health. However, there are elements that suggest that although the Ministry of Health agrees and concede power, they also express some concerns and can exert a certain amount of influence.

*“I believe it’s a good idea. Of course, there are ‘pros’ and ‘cons’”. (IV 25 Ministry of Health)*

*“So based on the religious decree they put it as compulsory. But we said that ok if you put it as compulsory, there should be some conditions”. (IV 31 Ministry of Health)*

*“Because to me, personally, whether you make it compulsory or voluntarily if you don’t provide good counselling, then it will give no meaning, whether you do it on a voluntary basis or compulsory basis. Number two, after counselling you have to keep your word; if you say ok, I will do it for you, then you have to do it. I mean in term of follow-up and support”. (IV 31 Ministry of Health)*

The caveat stipulated by the Ministry of Health is that with pre-marital testing there must also be pre and post-test counselling, which is in accordance with standard HIV guidelines and practice. This is one of the arguments against ‘mandatory’ HIV testing, that the focus is on the result and less on the process and raised by the participant below.

*“But its fine so long as they advise the couple pre-test counselling and all, they can actually adjust, be able to adjust to the implications of the results which they might get. So that’s very, very important on how they do. Otherwise, there could be issues”. (IV 11 Ministry of Health)”*

Of note, although some have mentioned that the practice could be extended to non- Muslims this has not been actualised, as yet.

*“It should be voluntary, there should be more awareness; it should be a decision made between the couples”. (IV 11 Ministry of Health)*

In addition, it was evident that some officials from the Ministry of Health perceived pre-marital HIV screening as akin to any other screening which was offered.

*“Well, it is just like other screening that we do”. (IV 31 Ministry of Health)*

In a sense, this is indicative of how premarital HIV testing has possibly become normalised in Malaysian society.

## People living with HIV

Participants living with HIV described support for premarital HIV testing, especially in terms of knowing one’s status and awareness.

*“[Premarital testing] It’s the best way to know your status; whether you are HIV positive or negative, by doing a screening test. It’s good for you and you can take care of yourself and maybe to your family or your partners”. (IV 14 PLHIV)*

One participant, a middle-aged housewife living with HIV, who believed she contracted the disease from her husband had the perception that perhaps having had the test could have prevented her diagnosis if she had been given the opportunity to be tested. The following is the lady’s statement translated from Malay during the interview by a representative from within the PLHIV Non-Government Organisation.

*“Ok, she never do the test, she say it’s very good from getting the awareness”. (IV28 PLHIV)*

Other participants who were living with HIV, expressed similar sentiments as the one described above, especially given that being a woman in Malaysia made them feel vulnerable to acquiring HIV. Despite this, there were concerns from some participants that premarital HIV testing predominated in a compulsory fashion in a region of low uptake of HIV testing, rather than encouraging a culture of voluntary HIV testing, described in the statement below.

*“Sometimes we need to make people aware that this is an involuntary kind of test; they need to have themselves want to go for the testing”. (IV 15 PLHIV)*

Although premarital HIV testing was supported it was also seen as providing a false sense of security, explains one participant.

*“Premarital HIV testing is quite tricky”. (IV 18 PLHIV)*

Elaborating further, that if after testing the couple are both negative, an impression that they will be *“safe forever”* is created, which is not necessarily the case adding that, *“premarital testing is not just a one stop shop”.*

There is also genuine concern that the mandatory nature of the testing, creates a sense of fear, amongst those who know they are HIV-positive, couples who:

*“Want to get married, but they are afraid” (IV 14 PLHIV)*.

The participant elaborated further that such a climate of fear meant that those people who have HIV but want to marry and do not want their family to know about their status *“can do anything to get the result”.* Explaining further after probing he recalled an anecdotal story from one of their partner organisations, where one couple wanted to get married and knew he was HIV positive but used “another person’s blood for testing” by using a different identity card. He indicates that these cases are quite rare and only resorted to because the couple may want to get married but foresee that their family would not accept their choice if the status was known resulting in family problems.

This same participant also speculated whether the testing was genuinely confidential, illustrated in the statement below.

*“Supposedly, it’s confidential; only the Ministry of Health knows your result, not even the other family should know your status. But now the religious department want to know your status, to give approval whether you can marry or not”. (IV 14 PLHIV)*

A minority of participants had objections to premarital HIV testing and were well aware that some international organisations were against testing on a mandatory basis, such as one participant.

*“The WHO (World Health Organisation) says ‘no’ you can’t have mandatory premarital HIV testing, so the only way to get this done was to go through the state religious authority”. (IV PLHIV 23)*

The participant also explained how premarital HIV testing was more detrimental towards women who were tested positive than men, harming women much more than it actually harms men, with wider implications.

*“The guy dumps her immediately, so she is left with a positive diagnosis, no more fiancé, how to explain to the family and no support, no nothing, no right- that's a disaster”. Whereas if it were the other way round, the tendency was for the woman to carry on [with the marriage]”*

This statement is particularly significant because it illustrates vividly why some people are against premarital HIV, the social context and the possible vulnerable position of women in society.

## Discussion

This study suggests that the majority of participants interviewed deemed premarital HIV testing, in the Malaysian context, as a beneficial measure, with a number of participants living with HIV themselves, expressing regret that they had not had the test and the perceived protection and prevention from HIV it offered. Although the WHO, human rights organisations and academics in the international HIV community have strong reservations about mandatory premarital HIV testing, this is strikingly in contrast to the participants of this study whom strongly support such testing.

It is apparent from the study that Islamic beliefs are hugely significant and of influence in the development of pre-marital HIV testing and this is visible in the close collaboration between the Ministry of Health officials and the department of Religious Affairs. Religious leaders strongly advocated for premarital HIV testing under the remit of ‘protection’ in keeping with the Shariah, which is understandable given their role as guardians of the faith. However, one wonders whether some comments from religious leaders that place premarital HIV testing as a way to ensure a ‘HIV baby’ is not born is due to general stigma or due to a lack of knowledge of how HIV can be reduced from mother to child by ARV treatment and other available measures. The same question could be postulated for those religious leaders who cited premarital HIV as a measure to reduce transmission from one spouse to another. A recent study in Malaysia suggested knowledge of religious leaders of HIV transmission and prevention were lower compared to other stakeholder groups [21], so this may well be the case.

In addition, Islamic religious beliefs seem also to shape officials from the Ministry of Health’s views about premarital HIV testing, articulated by the participant who deemed the measure as preventing ‘social disharmony’. In many respects, this is to be expected given the religious background of participants which may frame the way they see HIV prevention, although one could argue that the overseeing of social harmony may be beyond the purview of public health practitioners.

It is worth mentioning how the labelling of the premarital HIV testing differs amongst participants, ‘voluntary’, ‘compulsory’, ‘mandatory’ are all used adding to the confusion and to some degree is a reflection of the lack of clarity of the policy in certain respects. Essentially, premarital HIV testing is voluntary for health purposes, but mandatory for marriage purposes, for Muslim couples only.

Furthermore, as raised by one of the participants living with HIV, if premarital HIV testing exists for the sole purpose of protecting health, why is it not extended to non-Muslims in Malaysia, in the same mandatory capacity? The answer is intricately complicated by the way in which the policy was implemented in the first instance and likely tied in with religion, race, politics and a state and federal system of governance in Malaysia.

There is an overwhelming paucity of scientific research available in relation to premarital HIV testing, however, some of the results accrued through this study are consistent with the limited data garnered by other researchers who found strong support and acceptance of HIV testing in certain contexts. For example, Ganczak’s work in the Arab peninsula which found that there was high social acceptability of HIV testing amongst young Emirates which was indicative of the ‘feeling of vulnerability to contracting the infection’ [7]. Although in such Muslim countries premarital sex is against Islam the acceptance of such a test acknowledged that some Muslims do engage in such activities and testing may be an entry point to serve to provide a platform to educate on HIV/sexual health issues [7]. Thus the attitudes expressed in the study, show that although Islam forbids sex before marriage, premarital testing is considered favourably, which perhaps exposes the need for some way to address the fact that prospective partners may have difficulty disclosing their HIV status to their partners. This is particularly relevant given that in certain Muslim countries and the Middle East there are strong social and cultural mores against premarital sex but that equally Islamic principles of restraining from sexual activity may not always be achievable [22, 23].

In addition, there are striking parallels in our results to that of Luginaah and colleagues who conducted qualitative research amongst Christian church leaders in Ghana [2]. Religious leaders cited similar reasons for instituting premarital HIV testing, with one participant from their study stating, “we worked to protect our future generations”, similar to participants from this study who cited lineage (*Hukm*) and Islam looking to the future. Furthermore, Luginaah and colleagues found that what was described as ‘voluntary’ premarital HIV testing was not truly voluntary and that there were issues of confidentiality amid a reluctance to test for HIV [3].

The study by Rennie and colleagues which undertook an ethical analysis of premarital HIV testing in Goma, Congo, isolated several ‘potential burdens of policy’ [24]. Firstly, the restriction on the ‘right to marry’ and this can manifest in an informal way, by creating fear and secondly, a loss of confidentiality and stigmatization of people living with HIV [24)]; one could argue that in the case of Malaysia, even if these fears are perceived and not actual, a potential burden is created.

The ideal of truly voluntary testing with pre and post- test counselling and confidentiality assured for pre-marital HIV tests is favoured over mandatory measures, of course and thus the need to promote a culture of greater uptake of HIV testing in general throughout the population in Malaysia, by increasing awareness and reducing the stigma surrounding HIV ought to be emphasized. In a more recent paper, Mankandan and Sutan discusses some of the human rights concerns within the Malaysian context, including the obstruction to marriage and lack of confidentiality associated with mandatory premarital HIV screening [25]. They highlight that confidentiality is more at risk of being breached when either party tests positive for HIV and test results are handled by a myriad of people, including those from health and religious authorities to family members and adding that small modifications such as limiting the number of staff involved in testing as well as having “designated health care staff” could have a great impact. As well as obviating the need for religious leaders to know the test results [25].

However, in this interim period, there needs to be a greater understanding of the pre-marital HIV testing policy in Malaysia which presently exists. This includes a clearer role of the Ministry of Health and Religious Affairs in regulating pre-marital testing, including demystifying the terms ‘mandatory’ and ‘voluntary’ to aid transparency and a clear documentation of the protocol and procedure made freely available. Furthermore, a greater onus should be placed on ensuring that pre-marital HIV testing is undertaken in conjunction with pre and post-test counselling services which are non-judgemental, professional and strictly confidential.

In many ways, the situation of premarital HIV testing in Malaysia has many similarities with some of the discussions relating to mandatory HIV that have gone on before on a wider international scale. The analysis undertaken by Bayer and Edington of the history, dynamics and outcome serves as a useful resource to “those faced with policy choices” as well as highlighting “complex and politically charged relationships evolving between public health and human rights” [26]. The tensions between public health and human rights described in their analysis mirror some of the opinions and views that emerge from our findings.

Firstly, the typical viewpoint that considerations regarding public health trump concerns regarding human rights and that by having premarital mandatory HIV testing one also serves to normalise HIV testing per se.

Secondly, the atypical viewpoint, whereby human rights had to be safeguarded due to the potential consequences of paternalism and coerciveness [26]. Such examples are evident in some of the participant statements relating to prevention of “social disharmony”.

Thirdly, that these deliberations between matters of public health versus human rights are often heavily shaped by political, social and cultural contexts.

For example, in the Malaysian context, justification of premarital HIV testing is often attributed to the notion that such testing protects women, given the greater vulnerability and power of women in Malaysian society. In addition, considerations of health reigned supreme in comparison to those of rights perhaps because the idea of promoting and protecting health is valued more. Also that the modern day concept of human rights is decidedly Western in origin [27], whereas in Islamic jurisprudence there is the greater onus of social justice, equity and fairness [28]. Furthermore, the social and cultural context of Malaysia is shaped profoundly by Islam being the predominant religion in Malaysia, which shapes policy [29], process and also law [30], as evident in the dual legal system present in Malaysia.

## Limitations

Firstly, there was a certain degree of selection bias from within the PLHIV group, given that participants were recruited through the local NGO. Secondly, on a few occasions, there were difficulties with language amongst some participants who were more comfortable speaking in Malay than in English. This shortcoming was overcome by translation during the interview by colleagues of the participants’ interviewed from within the department or office, in the absence of financial resources for a professional translator.

Thirdly, there were limitations of time as well as the highly sensitive nature of the study.

## Conclusion

Although mandatory premarital HIV screening is considered contentious by many international organisations and human rights bodies and while the authors argue that HIV screening should operate in a voluntary capacity, fully confidential, with counselling; that ideal has not yet come to fruition in many countries to such an extent that premarital HIV testing is seen as an appropriate prevention strategy. Malaysia has implemented premarital HIV testing in response to rising levels of HIV, amidst a cultural and religious backdrop which may not provide the ideal enabling environment in which people can freely access services to test and know their HIV status. The findings of this study show that there is strong support for mandatory premarital HIV testing amongst participants, which could possibly expose the vulnerability to HIV, reluctance to test and other areas in the HIV response in Malaysia that need to be addressed, discussed and researched more fully.

## Abbreviations

ARV: AntiRetroViral
MSM: Men who have Sex with Men
PLHIV: People living with HIV
PMCT: Prevention of mother to child transmission
SW: Sex workers
TG: Transgender
UNAIDS: United Nations Programme on HIV and AIDS
UNHCR: United Nations High Commissioner for Refugees
WHO: World Health Organisation
